# Spirochetes in the Liver: An Unusual Presentation of a Common STI

**DOI:** 10.1155/2019/1012405

**Published:** 2019-12-11

**Authors:** Natasha Narang, Layth Al-Jashaami, Nayan Patel

**Affiliations:** ^1^Department of Internal Medicine, Banner University Medical Center, Phoenix, AZ, USA; ^2^Department of Gastroenterology, Banner University Medical Center, Phoenix, AZ, USA; ^3^Banner Transplant Institute, Phoenix, AZ, USA

## Abstract

It is estimated that 10% of patients with secondary syphilis have liver enzyme elevations, but clinical hepatitis is rare. However, in HIV-positive patients, syphilitic hepatitis may be much more common. We report a case of a 67-year-old male who developed progressively elevated liver enzymes, followed by development of neurological symptoms and then rash. Though the timeline of his symptom development was unusual, his constellation of symptoms prompted an RPR and FTA-ABS which returned reactive. He was additionally found to be HIV positive with a CD4 count of 946. He was treated with IV Penicillin, and his hepatitis improved thereafter.

## 1. Introduction

Often referred to as “the great imitator,” syphilis continues to be one of the most common sexually transmitted infections to this day, with a substantial proportion occurring in men who have sex with men [1]. The HIV population is particularly susceptible, with estimated incidence of up to 1/4 of all cases occurring in coinfected persons [2]. Though the separate phases of syphilis are well defined, clinical manifestations can greatly vary. It most commonly presents as painless genital ulcerations in the primary stage and progresses to rash, fever, and lymphadenopathy in the second [1, 2]. If secondary (disseminated) syphilis remains untreated, disease can span years and affect numerous organ systems. It has been reported that syphilis may affect mucocutaneous, gastrointestinal, pulmonary, renal, neurologic, and hepatic systems [1, 3]. When secondary syphilis signs recede, the patient enters the latent phase in which serologies remain positive without any overt signs or symptoms of infection. Tertiary syphilis may develop in about one-third of those with latent disease, presenting as neurosyphilis, aortic root insufficiency, or gummatous lesions [1, 4]. Overall, tertiary syphilis is scarce in our postantibiotic era.

## 2. Case Report

A 67-year-old male was admitted for progressive liver enzyme elevation. His symptoms began three months prior to this admission, when he presented to the emergency department with fatigue, decreased appetite, and abdominal pain and was found to have elevated transaminases. Initial evaluation by his outpatient gastroenterologist including workup for viral hepatitis, alpha-1 antitrypsin deficiency, primary biliary cirrhosis, Wilson's disease, and autoimmune hepatitis was largely inconclusive. Subsequently, he developed weakness and numbness that began distal to his axillae and progressed to his torso and lower extremities. The lower extremity symptoms worsened, and he developed ataxia, requiring a walker for ambulation. Two months after symptoms began, he underwent neurological workup for ataxia, right-sided weakness, and sporadic severe radiating low back pain. Imaging of his head, brain, and spine was unremarkable. On presentation, he attested to anorexia, 18 lb weight loss, weakness, lower extremity edema, “rusty” colored urine, and frequent episodes of “sharp” pain in his back, groin, and legs lasting minutes to hours. He also identified a nonitchy painless rash that began ten days prior on his arms and then spread to his torso, palms, and thighs. Past medical history was noncontributory He denied use of alcohol, tobacco, or drugs. He admitted to being sexually active with 5–10 male partners in the past year. There was no recent international travel or sick contacts and no use of antibiotics or herbal supplements.

**Figure 1:**
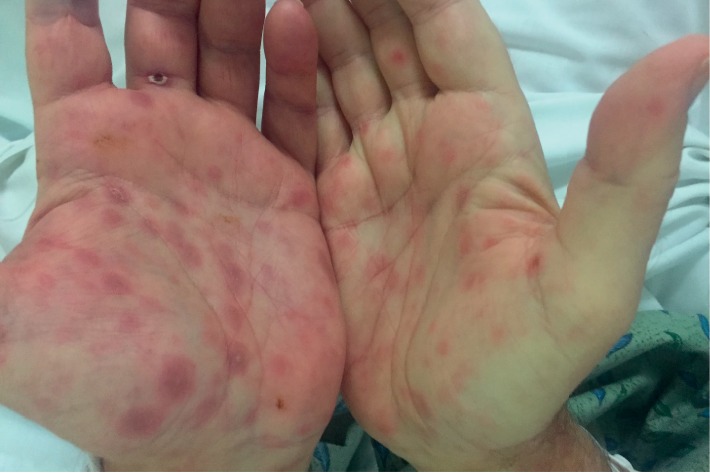
Palmar rash.

**Figure 2:**
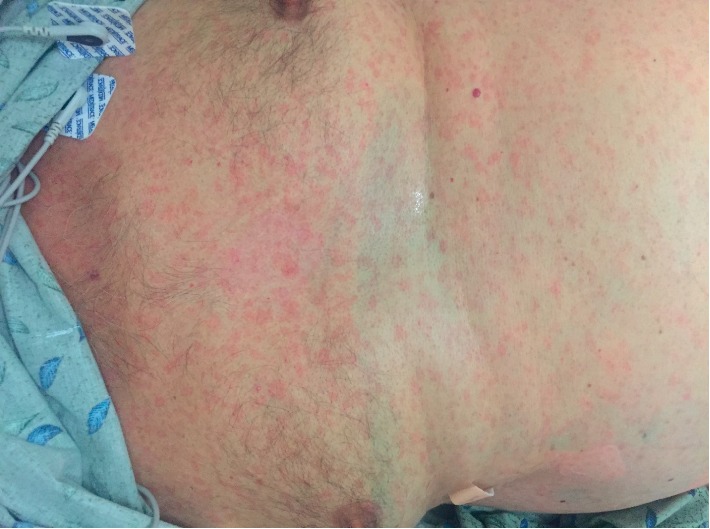
Trunk and abdominal rash.

On physical examination, he had mild scleral icterus, bilateral pitting lower extremity edema, and diminished sensation to pinprick and light touch in his bilateral lower extremities. His skin had a nontender maculopapular rash, most notable on the palms, thighs, chest, and scalp (Figures 1 and 2). A 1-2 cm nontender chancre was found on the posterior penile shaft.

Admitting labs were significant for total bilirubin 5.9, AST 201, ALT 116, and alkaline phosphatase 1048. Abdominal CT scan showed hepatomegaly with heterogeneous attenuation, patent hepatic vasculature, no focal lesions, and mild splenomegaly. HIDA scan showed patent cystic and common bile ducts. MRCP showed no extrahepatic biliary obstruction. Liver biopsy was performed.

**Figure 3:**
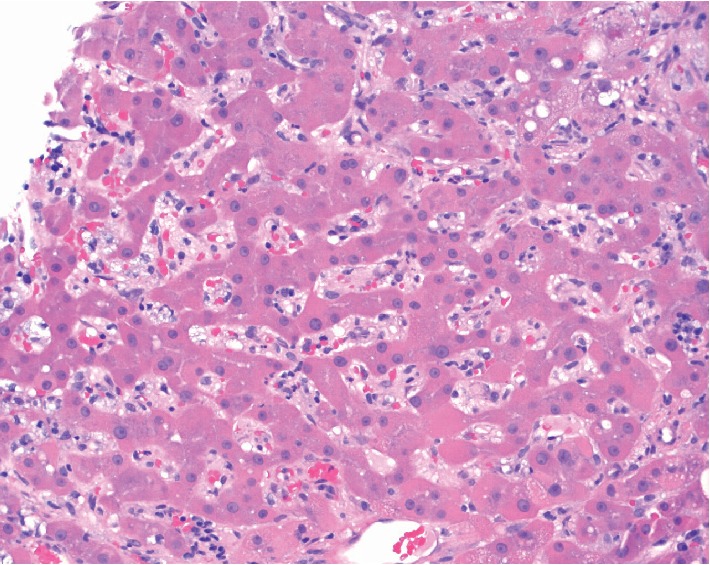
Immunohistochemistry for syphilis highlighting the organisms in sinusoidal, hepatocyte, and biliary epithelial cells.

**Figure 4:**
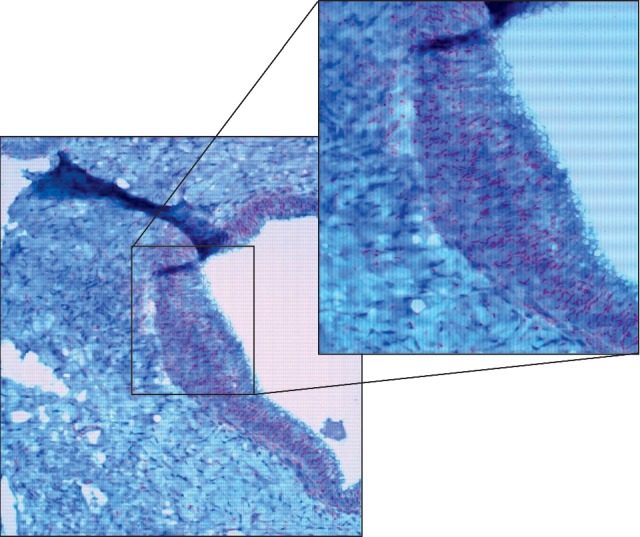
Treponemal immunostain of the large septal bile duct.

The coexistence of dermatologic, neurologic, and hepatic signs and symptoms prompted evaluation for syphilis. The patient had a reactive RPR titer of 1 : 256, reactive TPPA, and syphilis total antibody ratio of 15.8. Additionally, HIV screening was positive with a viral load of 650,493 copies/mL and CD4+ count of 946 cells/mm^3^. Liver pathology showed macrovesicular and microvesicular steatosis with focal hepatocellular ballooning and Mallory–Denk bodies, patchy PAS-D positive cytoplasmic hyaline globules, and periportal and sinusoidal fibrosis. Diagnosis of syphilitic hepatitis was confirmed by immunostain showing numerous treponemal spirochetes (Figures 3 and 4). A lumbar puncture was performed and showed a cell count of 7, nonreactive CSF VDRL titer, protein of 55 mg/dL, and glucose of 85 mg/dL, thus ruling out neurosyphilis. He was started on Penicillin G, and his liver enzymes improved impressively (Table 1).

## 3. Discussion

The first recognized case of hepatitis attributable to syphilis was reported in 1585 and termed “luetic jaundice” [4]. While syphilitic hepatitis has since been an established diagnosis in the medical literature as a component of secondary syphilis, it is not a commonly encountered etiology in patients seen for transaminitis, much less clinical hepatitis [5]. Cases that have been reported contain variable presentations including jaundice, dark urine, arthralgias, and generalized weakness [6].

Hepatic involvement has been characteristically described as a cholestatic pattern of injury with disproportionately elevated alkaline phosphatase compared to transaminases [1, 3, 7]. The preferential elevation of alkaline phosphatase is suspected to be due to pericholangiolar inflammation [2, 5]. Histologically, syphilitic hepatitis is visualized as inflammatory percolation in the portal region stimulating intralobular bile duct collapse and hepatocellular periportal necrosis [1]. Our patient had the distinguishing liver enzyme abnormalities plus the specific pathology findings, both diagnostic for syphilitic hepatitis. Additionally, the liver biopsy sampling showed numerous treponemes, a finding that is variable and relatively infrequent among published reports of syphilitic hepatitis [3].

Liver involvement in secondary syphilis is especially prevalent in patients with concurrent HIV infection, likely due to similar risk factors and degree of immunosuppression [2, 8]. The notable high rates of coinfection can be attributed to parallel risk factors including unprotected sexual activity, men who have sex with men, and intravenous drug use [4]. The most current CDC STD treatment guidelines emphasize the importance of routine HIV screening in all patients who pursue evaluation and therapy for any STDs [9]. The patient discussed in this case was immediately screened for additional sexually transmitted infections when the syphilis diagnosis was made, resulting in the discovery of his HIV-positive status. A case study and review performed by Mullick et al. identified a linear relationship between RPR titer and absolute CD4+ T-lymphocytes count [8]. This supports a presumption that clinical manifestations of hepatitis due to syphilitic periportal inflammation is more likely to be apparent in those with preserved host inflammatory response.

The causative role of *Treponema pallidum* in hepatocellular damage is supported by the resolution of laboratory and clinical aberrations following treatment with intramuscular or intravenous Penicillin G [8]. Thus, it is important that early identification of this infrequent presentation of syphilis is made because of its easy reversibility and subsequent prevention of progression to further stages. This case additionally emphasizes the importance for ensuring infectious etiologies remain in the differential diagnoses of elevated liver function tests.

## Figures and Tables

**Table 1:** Pertinent labs prior to and after treatment.

Days from treatment	AST (IU/L)	ALT (IU/L)	Alkaline phosphatase (IU/L)	Total bilirubin (mg/dL)	PLT	INR
*T* − 4	193	118	1074	5.9	171	1.6
*T* − 3	201	126	1144	9.1	166	1.7
*T*	330	125	1149	8.7	148	1.8
*T* + 4	277	120	835	8.3	137	1.6
*T* + 7	226	116	866	8.0	153	1.6
*T* + 12	217	127	894	4.4	159	1.6
*T* + 16	208	119	816	3.3	163	1.6

*T*  =  day of penicillin treatment initiation.
